# Effect of metformin on maternal and fetal outcomes in obese pregnant women (EMPOWaR): a randomised, double-blind, placebo-controlled trial

**DOI:** 10.1016/S2213-8587(15)00219-3

**Published:** 2015-10

**Authors:** Carolyn Chiswick, Rebecca M Reynolds, Fiona Denison, Amanda J Drake, Shareen Forbes, David E Newby, Brian R Walker, Siobhan Quenby, Susan Wray, Andrew Weeks, Hany Lashen, Aryelly Rodriguez, Gordon Murray, Sonia Whyte, Jane E Norman

**Affiliations:** a Tommy's Centre for Maternal and Fetal Health, Medical Research Council (MRC) Centre for Reproductive Health, Queen's Medical Research Institute, Edinburgh, UK; b British Heart Foundation Centre for Cardiovascular Science, Queen's Medical Research Institute, Edinburgh, UK; c Chancellor's Building, Royal Infirmary of Edinburgh, Edinburgh, UK; d Division of Reproductive Health, Warwick Medical School, University of Warwick, Coventry, UK; e Faculty of Health and Life Sciences, First Floor, Liverpool Women's Hospital, Liverpool, UK; f Academic Unit of Reproductive and Developmental Medicine, The Jessop Wing, Sheffield, UK; g Centre for Population Health Sciences, Teviot Place, Edinburgh, UK

## Abstract

**Background:**

Maternal obesity is associated with increased birthweight, and obesity and premature mortality in adult offspring. The mechanism by which maternal obesity leads to these outcomes is not well understood, but maternal hyperglycaemia and insulin resistance are both implicated. We aimed to establish whether the insulin sensitising drug metformin improves maternal and fetal outcomes in obese pregnant women without diabetes.

**Methods:**

We did this randomised, double-blind, placebo-controlled trial in antenatal clinics at 15 National Health Service hospitals in the UK. Pregnant women (aged ≥16 years) between 12 and 16 weeks' gestation who had a BMI of 30 kg/m^2^ or more and normal glucose tolerance were randomly assigned (1:1), via a web-based computer-generated block randomisation procedure (block size of two to four), to receive oral metformin 500 mg (increasing to a maximum of 2500 mg) or matched placebo daily from between 12 and 16 weeks' gestation until delivery of the baby. Randomisation was stratified by study site and BMI band (30–39 *vs* ≥40 kg/m^2^). Participants, caregivers, and study personnel were masked to treatment assignment. The primary outcome was *Z* score corresponding to the gestational age, parity, and sex-standardised birthweight percentile of liveborn babies delivered at 24 weeks or more of gestation. We did analysis by modified intention to treat. This trial is registered, ISRCTN number 51279843.

**Findings:**

Between Feb 3, 2011, and Jan 16, 2014, inclusive, we randomly assigned 449 women to either placebo (n=223) or metformin (n=226), of whom 434 (97%) were included in the final modified intention-to-treat analysis. Mean birthweight at delivery was 3463 g (SD 660) in the placebo group and 3462 g (548) in the metformin group. The estimated effect size of metformin on the primary outcome was non-significant (adjusted mean difference −0·029, 95% CI −0·217 to 0·158; p=0·7597). The difference in the number of women reporting the combined adverse outcome of miscarriage, termination of pregnancy, stillbirth, or neonatal death in the metformin group (n=7) versus the placebo group (n=2) was not significant (odds ratio 3·60, 95% CI 0·74–17·50; p=0·11).

**Interpretation:**

Metformin has no significant effect on birthweight percentile in obese pregnant women. Further follow-up of babies born to mothers in the EMPOWaR study will identify longer-term outcomes of metformin in this population; in the meantime, metformin should not be used to improve pregnancy outcomes in obese women without diabetes.

**Funding:**

The Efficacy and Mechanism Evaluation (EME) Programme, a Medical Research Council and National Institute for Health Research partnership.

## Introduction

The adverse effects of maternal obesity on short- term pregnancy complications include pre-eclampsia,^1^ caesarean section, increased duration of maternal and neonatal hospital stay, maternal haemorrhage, infant mortality,^2^ and stillbirth.^3^ Maternal obesity during pregnancy is also associated with raised birthweight and neonatal fat mass.^3^, ^4^

Accumulating data suggest that maternal obesity might predispose offspring to later life obesity, with high birthweight being a marker for increased risk. Correlations between high birthweight and adult obesity have been reported in large epidemiological studies,^5^, ^6^ a systematic review,^7^ and a validated prediction model.^8^ The rapid rise in the prevalence of both high birthweight^9^ and maternal obesity mean that their links with later life obesity are a major concern. Indeed, in a record linkage study,^10^ we showed that maternal obesity was associated with a 35% increase in the hazard of all-cause offspring mortality in adulthood, even after adjustment for confounders. As such, an effective intervention applied during pregnancy could have a major effect on interruption of the cycle of maternal obesity and offspring obesity and ill health, thus helping to reverse the upward secular trend in obesity prevalence.

Much evidence implicates insulin resistance (ie, when a defined concentration of insulin does not effect a predictable metabolic response) and hyperglycaemia as the mechanism by which maternal obesity causes excessive neonatal birthweight. Obese pregnant women are significantly more insulin resistant and hyperglycaemic than are pregnant women of a normal weight,^11^ and several large studies, including the Camden study^12^ and the HAPO study,^13^ show a positive association between high glucose concentrations and macrosomia, even at glucose concentrations regarded as normal during pregnancy. Additionally, a Cochrane review protocol^14^ has outlined additional potential benefits on mother and baby of metformin in obese pregnant women.

**Research in context**

**Evidence before this study**

We searched Medline between Jan 1, 1980, and April 30, 2015, with the terms “metformin”, “pregnancy”, “birthweight”, and “randomised trial”. Four reports were identified: three focused on women with gestational diabetes (ie, a different study population to our study) and one was the protocol for the EMPOWaR study. Birthweight was a secondary outcome in one study (Vanky et al, 2010), in which pregnant women with a history of polycystic ovary syndrome were randomly assigned to receive metformin or placebo.

**Added value of this study**

To our knowledge, this is the first placebo-controlled study designed to establish the effect of metformin on birthweight in obese pregnant women.

**Implications of all the available evidence**

Metformin given to normally glucose tolerant obese pregnant women from 12– 16 weeks' gestation until delivery has no significant effect on gestational age, parity, or sex-standardised birthweight percentile. Further follow-up of babies born to mothers in the EMPOWaR study will identify longer-term outcomes of metformin in this population. In the meantime, metformin should not be used to improve pregnancy outcomes in obese women without diabetes.

In view of these findings, we did this EMPOWaR study^15^ to test the hypothesis that the insulin sensitising drug metformin would reduce birthweight when given to obese women during pregnancy. On the basis of findings from other epidemiological studies^5^, ^6^, ^16^ a reduction in birthweight would be expected to result in a reduction in future life risk of obesity and metabolic syndrome in the offspring.

## Methods

### Study design and participants

We did this randomised, double-blind, placebo-controlled trial in antenatal clinics at 15 National Health Service (NHS) hospitals in the UK. Eligible women were aged 16 years or older, had a BMI of 30 kg/m^2^ or more, and were between 12 and 16 weeks' gestation. We excluded non-white women and those with: pre-existing diabetes; gestational diabetes in a previous pregnancy; gestational diabetes diagnosed in the index pregnancy before randomisation; systemic disease at the time of trial entry (requiring either regular drugs or treatment with systemic corticosteroids in the past 3 months); previous delivery of a baby smaller than the 3rd percentile for weight; previous pregnancy with pre-eclampsia prompting delivery before 32 weeks' gestation; known hypersensitivity to metformin hydrochloride or any of the excipients; known liver failure; known renal failure; acute disorders at the time of trial entry with the potential to change renal function, such as dehydration sufficient to require intravenous infusion, severe infection, shock, intravascular administration of iodinated contrast agents, or acute or chronic diseases that might cause tissue hypoxia (eg, cardiac or respiratory failure, recent myocardial infarction, hepatic insufficiency, acute alcohol intoxication, or alcoholism); lactating women; and women with multiple pregnancy.

The study was approved by the Scotland A research ethics committee (reference number 10/MRE00/12) and the Medicines and Healthcare products Regulatory Agency (EudraCT number 2009-017134-47). All participants provided written information consent. The protocol has been published elsewhere^15^ and is available online.

### Randomisation and masking

We randomly assigned participants (1:1), via a web-based computer-generated block randomisation procedure (block size of two to four), to receive metformin or placebo. Randomisation was stratified by study site and BMI band (30–39 *vs* ≥40 kg/m^2^). Participants, caregivers, and study personnel were masked to treatment assignment. Members of the independent Data Monitoring Committee had access to unmasked data reports, but had no contact with study participants.

### Procedures

Demographics, medical history, and maternal anthropometry were recorded at baseline. A formal 75 g oral glucose tolerance test was done in addition to screening for liver and renal function. We excluded participants with impaired renal function (urea >6·6 mmol/L, creatinine >85 μmol/L, sodium >145 mmol/L, potassium >5·0 mmol/L), or liver function (bilirubin >16 μmol/L, alanine transferase >60 IU/L), or with abnormal lactate (according to local laboratory reference range) or gestational diabetes defined by WHO criteria (fasting glucose ≥7·0 mmol/L and 2 h glucose ≥7·8 mmol/L), or any other local hospital criteria (eg, International Association of Diabetes and Pregnancy Study Groups [IADPSG]^17^).

Participants received oral metformin 500 mg or matched placebo tablets, in a dose of up to five tablets daily in two to three divided doses. Treatment was initiated at 12–16 weeks' gestation and continued until delivery of the baby. Treatment started at one 500 mg tablet once a day at week 1, and escalated by one tablet a day each week over 5 weeks, to reach either the maximum tolerable dose or the maximum permitted dose of 2500 mg, whichever was lower. In the case of side-effects, participants were advised to reduce the current dose to that of the previous week, and wait for 1 week before increasing the dose again. The local investigator was allowed to change the treatment regimen at their discretion, as long as the maximum daily dose did not exceed 2500 mg in three divided doses. Participants were asked to keep a diary of drug intake and to bring all drugs to each study visit to monitor compliance.

Randomised participants were reviewed face to face or by telephone at 18–20, 28, 36, and 40 weeks' gestation; around the time of delivery; and 3 months postnatally. Pregnancy complications were recorded and women were asked to complete a side-effect questionnaire at each review visit until delivery. Maternal anthropometry was repeated at 36 weeks' gestation and 3 months postnatally. The glucose tolerance test was repeated at 28 and 36 weeks' gestation, and blood was stored for measurement of inflammatory and metabolic indices. The protocol recommended that women who developed gestational diabetes should be given insulin whilst maintaining study treatment and blinding. The baby's weight and anthropometry were recorded at delivery and at the 3 month postnatal visit.

### Outcomes

The primary outcome was *Z* score corresponding to the gestational age, parity, and sex-standardised birthweight percentile of liveborn babies delivered at 24 or more weeks' gestation. The main secondary outcome was maternal insulin resistance at 36 weeks' gestation. Other secondary outcomes included maternal fasting glucose and insulin and 2 h glucose at 36 weeks; maternal anthropometry and body composition; baby anthropometry and body composition; maternal inflammatory and metabolic outcomes at 36 weeks, including C-reactive protein (CRP), cholesterol, HDL, LDL, triglycerides, interleukin (IL)-6, leptin, serum cortisol, non-esterified fatty acids, and the ratio of plasminogen activator inhibitor 1 to 2; incidence of low birthweight percentile (<3rd and <10th); incidence of other adverse maternal and neonatal outcomes, including maternal symptoms; maternal plasma metformin concentration to explore tablet taking in the metformin group; and the maternal metabolic (fasting glucose and insulin and 2 h glucose) and inflammatory markers at 28 weeks. The methods for detection of the blood analytes have been described elsewhere.^15^ Secondary mechanistic outcomes as outlined in the published protocol^15^ were obtained in a subset of participants and will be reported elsewhere.

We made some changes to the protocol after recruitment began, but before generation of the statistical analysis plan, publication of the protocol,^15^ and unmasking and analysis. Specifically, maternal insulin resistance at 36 weeks' gestation was originally a co-primary outcome, but was relegated to a secondary outcome when a substantial proportion of participants did not provide a blood sample at 36 weeks. Additionally, we used patient self-reporting of tablet taking to establish treatment compliance and inform the per-protocol analysis.

### Statistical analysis

We calculated that a sample size of 143 women in each group would provide 80% power, and a sample size of 163 women in each group would provide 85% power, to detect a difference in mean birthweight percentile of SD 0·33 (equivalent to the difference between a placebo mean of 4·0 kg^18^ and a metformin mean of 3·8 kg) at a two-sided 5% significance level with a two-group *t* test. We initially aimed to randomise 400 women based on anticipated high compliance and follow-up rates, but in a protocol amendment increased our sample size to 450 women when anecdotal evidence (without formal testing) suggested that compliance was lower than anticipated.

We did our primary analysis in the modified intention-to-treat population. We also did per-protocol analyses, in which we compared outcomes amongst participants who were compliant with treatment. Compliance was determined before review of the data or unmasking. To measure compliance we calculated the number of weeks from randomisation to delivery for each woman; participants reporting (via their study diary) that they took at least one tablet on at least 4 days per week for at least half of those weeks were deemed to have been compliant. We did not use plasma metformin to measure compliance as no such measure of compliance could be done for placebo.

We did exploratory analyses of secondary outcomes. No formal adjustment was made to any p values to allow for the large number of secondary endpoints analysed, and thus p values for secondary analyses need to be interpreted conservatively. We also did post-hoc analyses of safety outcomes of all reported serious adverse events and the combined adverse outcome of stillbirth, neonatal death, termination of pregnancy, or miscarriage.

We derived birthweight percentiles and *Z* scores of birthweight percentiles (livebirths only) for each patient after adjustment for sex, gestational age, and parity (nulliparous *vs* multiparous) with population-derived charts.^19^ We used a linear regression model adjusted for treatment centre and BMI band (30–39 *vs* ≥40 kg/m^2^) to compare *Z* scores between the groups and to obtain the adjusted mean difference with 95% CI. This method was also used for other continuous outcomes including glucose and insulin and homeostatic model assessment of insulin resistance (HOMA-IR). When necessary, we did log transformations to achieve normal distribution of data before statistical testing. For assessment of CRP concentration in the umbilical cord, we used Kruskal–Wallis one-way analysis of variance because this variable could not be transformed into a normal distribution. We used unadjusted logistic regression for binary outcomes and Fisher's exact test when the event counts were small. Relevant denominators were either all participants randomised for whom information was available, or those having a livebirth for whom information was available.

We did analyses with SAS (version number 9.3). A trial steering and a data and safety monitoring committee oversaw the study. The trial was registered, ISRCTN number 51279843.

### Role of the funding source

The funder of the study had no role in study design, data collection, data analysis, data interpretation, or writing of the report. The corresponding author had full access to all the data in the study and had final responsibility for the decision to submit for publication.

## Results

Between Feb 3, 2011, and Jan 16, 2014, inclusive, we randomly assigned 449 participants to the placebo group (n=223) or the metformin group (n=226), of whom 434 (97%) were included in the modified intention-to-treat analysis (figure). The most common reasons for non-participation were a concern that study drugs might be harmful to the baby, and low awareness about the adverse effects of obesity on pregnancy outcome. Baseline demographics, medical history, and maternal anthropometry were similar between groups (table 1).

From diary returns and analysis with predefined criteria, 118 (67%) of 177 women in the placebo group and 109 (65%) of 167 women in the metformin group were deemed compliant. Subsequent analysis of metformin concentrations showed that detectable concentrations were present in the blood of 80 (61%) of 131 women in the metformin group who gave a blood sample at 36 weeks' gestation. To explore dosage, we identified the proportion of drug-taking days when 2500 mg or 2000 mg of study drug was taken. In the placebo group, for 56% of all possible tablet-taking days, the top dose of 2500 mg was taken, and for 68% of these days a dose of 2000 mg or more was taken; the corresponding values in the metformin group were 38% and 62%, respectively.

Mean birthweight at delivery was 3463 g (SD 660) in the placebo group and 3462 g (548) in the metformin group (table 2). Mean birthweight percentile was high in both groups (table 2); the proportion of liveborn babies weighing more than the 90th percentile was similar between the placebo group and the metformin group (38 [17%] of 220 and 31 [14%] of 214 babies, respectively). The primary outcome of *Z* score of birthweight percentile for babies liveborn at 24 weeks or more of gestation, standardised for sex, parity and gestation at delivery, was similar between the metformin and placebo groups, and the estimated effect size of metformin on the primary outcome was non-significant (table 2).

We recorded no evidence of a reduction in the main secondary outcome of HOMA-IR at 36 weeks' gestation, nor any evidence of a clinically or statistically significant effect of metformin on fasting or 2 h glucose (after a 75 g oral glucose challenge) or fasting insulin at 36 weeks' gestation (table 3). By contrast, fasting glucose and HOMA-IR score at 28 weeks' gestation was lower in women in the metformin group than in those in the placebo group (appendix). Metformin had no significant effect on the anthropometric variables of maternal weight gain in pregnancy or neonatal ponderal index (table 3).

Plasma IL-6 and CRP concentrations were both significantly lower in women given metformin, but no differences were shown in other biochemical outcomes (table 3). Metformin did not seem to prevent gestational diabetes, as proportions of women fulfilling either IADSPG (table 4) or WHO (data not shown) criteria for gestational diabetes at any time in pregnancy were similar between the two groups (table 4). Furthermore, metformin did not delay the onset of gestational diabetes (IADPSG criteria): 26 women in the placebo group were diagnosed at 28 weeks' gestation and ten women were diagnosed at 36 weeks compared with 11 women diagnosed at 28 weeks and 15 women at 36 weeks in the metformin group (p=0·0718, Mantel-Haenszel χ^2^; post-hoc analysis).

Maternal symptoms of diarrhoea and vomiting were more common in women in the metformin group (table 4). The incidence of other adverse outcomes, including preterm birth and low birthweight, caesarean section, and postpartum haemorrhage were similar in the two groups (table 4). We recorded no adverse effects of metformin in post-hoc safety analyses comparing the proportion of women with a recordable serious adverse event between the two groups (table 4). The increase in the combined adverse outcome of miscarriage, termination of pregnancy, stillbirth or neonatal death in women in the metformin group was not significant (table 2). Admission to the neonatal unit was less common in the metformin group than the placebo group (table 4). We noted no differences in outcomes at other timepoints between the two groups (appendix), with the exception of fasting glucose and HOMA-IR score, as mentioned above.

Further analyses of the data on a per-protocol basis resulted in similar findings to the modified intention-to-treat analysis, with the exception of vomiting and CRP concentration, in which the direction of differences was maintained but the results were no longer significant (appendix), and in 2 h glucose (estimated mean difference −0312 mmol/L, 95% CI −0·620 to −0·004; p=0·0471) and fasting insulin (6·04 pmol/L, 5·40–6·78; p=0·0173) at 28 weeks' gestation, which were significantly lower in the metformin group than in the placebo group.

## Discussion

To our knowledge, EMPOWaR is the first trial of a pharmacological intervention to reduce the risk of ill health in later life, using birthweight as a surrogate marker, in the offspring of obese pregnant women. By contrast with our original hypothesis, metformin given at a median dose of 2000 mg daily to obese and severely obese pregnant women (mean BMI 37·7 kg/m^2^) without diabetes, from 12–16 weeks' gestation until delivery, had no effect on birthweight or neonatal or maternal anthropometry. On the basis of the study being powered to detect a clinically meaningful effect size, we conclude that this finding shows a true absence of effect of metformin on birthweight rather than a type 2 error. The absence of effect was apparent in both intention-to-treat and per-protocol analyses. We conclude that metformin does not have a role in reducing the birthweight of offspring of obese pregnant women.

The strengths of this study are its multicentre randomised controlled design, making the study robust and generalisable, and that, despite women's natural reluctance to take medication during pregnancy, we were able to recruit to our target sample size, generating adequate power to address our hypothesis.

Although compliance was lower than anticipated, this was balanced by the SD for birthweight also being lower. As such, the 95% CI for the primary comparison in both the intention-to-treat and per-protocol analyses both exclude the prespecified minimum clinically relevant effect size of 0·33. We conclude that the failure to detect a significant difference between the groups is a strong negative finding rather than a result of the trial being underpowered.

Studies of other interventions aimed at reducing birthweight in obese pregnant women, including diet and lifestyle interventions,^20^, ^21^, ^22^ have likewise shown no significant effects. Our data showing that metformin has no effect on birthweight in obese and severely obese pregnant women are in line with secondary outcome data from a smaller study of metformin in non-obese (mean 29·5 kg/m^2^ [SD 7·0]) pregnant women with a history of polycystic ovary syndrome.^23^ We are aware of two other ongoing studies of the effect of metformin in obese pregnant women (Clinicaltrials.gov, number NCT01273584 and Australian New Zealand Clinical Trials registry, ACTRN 12612001277831).

We believe that metformin had its expected pharmacodynamic effects. Fasting glucose and insulin were lower in the metformin group than the placebo group at 28 weeks in the intention-to-treat analysis, and fasting and 2 h glucose, insulin, and HOMA-IR were lower in the metformin group at 28 weeks in the per-protocol analysis. The subsequent lack of effect of metformin at 36 weeks is initially surprising, but might indicate changes in glucose homoeostasis throughout pregnancy in obese women.

Although metformin had no effect on the primary outcome, the metformin-associated reduction in inflammatory markers CRP and IL-6 might be beneficial. These markers are found at higher concentrations in obese pregnant women than in pregnant women of a normal weight^19^ and have been associated with adverse outcomes such as preterm birth and pre-eclampsia.^24^, ^25^ Our findings are consistent with those in non-pregnant individuals, in whom metformin reduces concentrations of CRP^26^ and (variably) IL-6.^27^

Absence of efficacy of metformin in reducing mean birthweight, despite lowering maternal glucose and insulin in mid-pregnancy, casts doubt on the 1952 Pedersen hypothesis^28^ that maternal hyperglycaemia drives fetal hyperglycaemia, and hence fetal hyperinsulinaemia and fetal overgrowth. Other investigators have hypothesised, by contrast with Pedersen, that excess maternal lipids might be as, or even more important than, excess maternal glucose in fetal fat accumulation, particularly in the presence of maternal obesity.^29^ The present study provides the first experimental evidence that factors other than maternal glucose are important in fetal overgrowth, challenging conventional thinking about the factors linking maternal obesity and offspring macrosomia.

Metformin might have a beneficial effect on future life risk of obesity and metabolic syndrome in offspring, even in the absence of an effect on birthweight percentile. In an animal study,^30^ prenatal metformin improved glucose tolerance, and reduced accumulation of bodyweight, and fat mass in adulthood of the offspring, despite having only marginal effects on birthweight. Additionally, in the Metformin in Gestational diabetes (MiG) study,^31^ children of women randomised to the metformin group had lower visceral body fat at 2 years than did children of women randomised to insulin, despite similarities in birthweight. Further follow-up of babies born to mothers in the EMPOWaR trial is planned to explore this possibility and will identify longer-term outcomes on offspring of obese women given metformin in pregnancy. In the meantime, metformin should not be used to improve pregnancy outcomes in obese women.

## Figures and Tables

**Figure:**
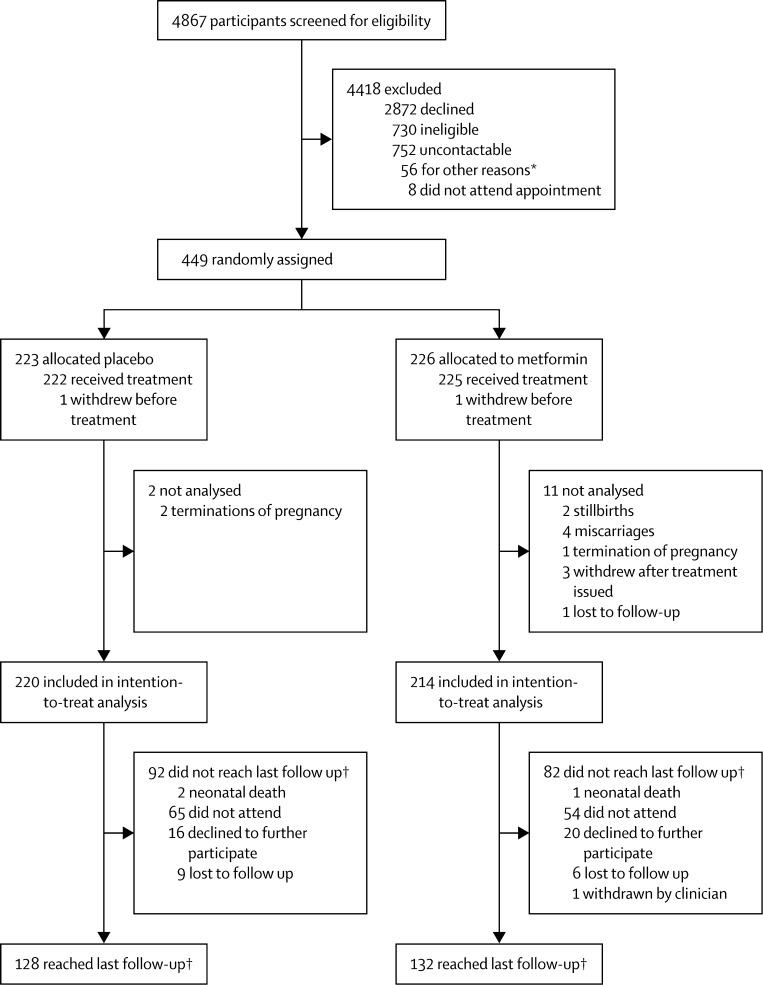
Trial profile *Change in eligibility from screening of notes to recruitment visit (unable to arrange recruitment visit before 16 weeks [n=26]), recruitment stopped before screening appointment (n=14), miscarriage (n=2), moved out of area (n=1), unable to provide informed consent because of difficulties with spoken English (n=5), own doctor or midwife advised against participation (n=4), duplicate note screening number issued in error (n=4). †3 months after birth.

**Table 1:** Baseline characteristics

		**Placebo group**		**Metformin group**	
		**Mean (SD) or n (%)**	**N**	**Mean (SD) or n (%)**	**N**
**Demographics and lifestyle**					
Age (years)		28·9 (5·1)	223	28·7 (5·8)	226
Currently smokes		31 (14%)	223	40 (18%)	226
Currently drinks alcohol		9 (4%)	223	3 (1%)	226
Illicit drug use		1 (<1%)	223	0	226
Highest educational qualification					
	School for ≤16 years	79 (35%)	223	75 (33%)	226
	School for ≥16 years	144 (65%)	223	151 (67%)	226
At least one previous pregnancy ≥12 weeks' gestation		161 (73%)	220	147 (65%)	226
Systolic blood pressure (mm Hg)		119·4 (10·4)	223	117·6 (10·8)	226
Diastolic blood pressure (mm Hg)		68·9 (7·3)	223	68·0 (7·8)	226
Gestation at baseline (days)		98·9 (8·7)	223	99·1 (8·1)	226
**Medical history**					
Pre-eclampsia or pregnancy induced hypertension		7 (3%)	223	10 (4%)	226
Pre-pregnancy hypertension requiring treatment		2 (1%)	223	1 (<1%)	226
Polycystic ovary syndrome		21 (9%)	223	28 (12%)	226
Depression requiring treatment		71 (32%)	223	48 (21%)	226
Anxiety requiring treatment		20 (9%)	223	15 (7%)	226
**Family history**					
Cardiovascular disease		69 (31%)	223	71 (31%)	226
Pre-eclampsia		22 (10%)	223	19 (8%)	226
Diabetes		101 (45%)	223	99 (44%)	226
Other		96 (43%)	223	109 (48%)	226
**Anthropometry**					
Height (cm)		165·1 (5·9)	223	165·5 (5·9)	226
Weight (kg)		102·9 (17·0)	223	103·6 (15·5)	226
BMI (kg/m^2^)		37·7 (5·6)	223	37·8 (4·9)	226
Waist (cm)		108·7 (13·5)	222	110·1 (11·9)	225
Hip (cm)		126·4 (12·1)	222	127·4 (11·8)	225
Mid-arm (cm)		36·3 (5·0)	220	36·7 (4·7)	221
Mid-thigh (cm)		64·1 (7·7)	219	64·2 (6·9)	222
Tricep skinfold (mm)		31·2 (9·7)	222	31·9 (10·8)	222
Bicep skinfold (mm)		25·7 (10·0)	222	27·4 (10·9)	222
Subscapular skinfold (mm)		32·0 (12·2)	222	32·6 (11·8)	220
Maternal fat (%)*		46·8 (5·6)	48	48·2 (5·2)	53
**Blood tests**					
Fasting glucose (mmol/L)		4·39 (0·34)	223	4·41 (0·40)	226
2 h glucose (mmol/L)†		5·50 (1·09)	223	5·20 (1·08)	226
Fasting insulin (pmol/L)		153·35 (70·84)	189	152·44 (85·15)	188
HOMA-IR score‡		4·36 (2·16)	189	4·36 (2·76)	188
C-reactive protein (mg/L)		11·1 (7·4)	221	10·7 (6·9)	223
Cholesterol (mmol/L)		4·87 (1·15)	216	4·88 (1·09)	214
HDL (mmol/L)		1·67 (0·39)	215	1·64 (0·38)	214
LDL (mmol/L)		2·91 (0·78)	194	2·89 (0·86)	191
Triglycerides (mmol/L)		1·51 (0·53)	216	1·43 (0·56)	214
Interleukin-6 (mmol/L)		2·77 (5·50)	189	2·63 (4·37)	188
Leptin (ng/mL)		93·6 (42·1)	189	98·5 (40·3)	188
Serum cortisol (nmol/L)		396·4 (143·6)	189	431·0 (178·8)	188
NEFA (mmol/L)		0·52 (0·20)	189	0·48 (0·18)	188
PAI-1 to PAI-2 ratio		1·48 (1·39)	131	1·77 (5·22)	128
**Putative father details**					
Height (cm)		178·5 (8·3)	204	177·1 (13·7)	202
Weight (kg)		92·3 (22·5)	187	93·5 (25·8)	188
Ethnic origin					
	White	214 (96%)	223	210 (94%)	224
	Mixed	4 (2%)	223	4 (2%)	224
	Asian	0	223	3 (1%)	224
	Black	4 (2%)	223	6 (3%)	224
	Chinese	0	223	0	224
	Other	1 (<1%)	223	1 (<1%)	224
(Table 1 continues on next page)					

HOMA-IR= homeostatic model assessment of insulin resistance. NEFA=non-esterified fatty acids. PAI=plasminogen activator inhibitor.

* Measured only in Edinburgh participants.

† After a 75 g oral glucose challenge.

‡ Fasting glucose (mmol/L) x insulin (μIU/L).

**Table 2:** Primary and birth outcomes

	**Placebo group**		**Metformin group**		**Adjusted mean difference or OR (95 % CI)**	**p value**
	**Mean (SD) or n (%)**	**N**	**Mean (SD) or n (%)**	**N**		
**Primary outcome**						
*Z* score of birthweight percentile*	0·2680 (1·0055)	220	0·2464 (1·0179)	214	−0·029 (−0·217 to 0·158)	0·76
**Birth outcome (all births)**						
Livebirth at ≥24 weeks' gestation	220 (99%)	222	214 (97%)	221	..	..
Stillbirth at ≥24 weeks' gestation, miscarriage, or termination of pregnancy	2 (1%)†	222	7 (3%)‡	221	3·597 (0·739 to 17·504)§	0·11
**Birth outcome (liveborn babies at ≥24 weeks' gestation)**						
Gestational age at delivery (days)	275·9 (15·9)	220	276·6 (11·7)	214	..	..
Male sex	109 (50%)	220	109 (51%)	214	..	..
Birthweight at delivery (g)	3463 (660)	220	3462 (548)	214	..	..
Birthweight percentile	57·3 (27·9)	220	56·9 (28·6)	214	..	..

OR=odds ratio.

* Percentile by gestational age, sex, and parity for livebirths at ≥24 weeks' gestation.

† Two terminations of pregnancy, one for fetal abnormality (split hand and foot syndrome) and one after a spontaneous membrane rupture at 18 weeks' gestation.

‡ Of the two stillbirths, one was at 31 weeks of a baby with a known cardiac anomaly and severe hydrops fetalis and one was an intrauterine death of a normally formed baby born at 38 weeks with a birthweight less than the third percentile for gestation. Of the four miscarriages, one was after a road traffic accident and three were spontaneous. One termination of pregnancy was done after a diagnosis of trisomy 21. None of the women returned diaries nor provided a blood sample for analysis of metformin.

§ OR from post-hoc analysis.

**Table 3:** Secondary outcomes

	**Placebo group**		**Metformin group**		**Adjusted mean difference or ratio (95% CI)**	**p value**
	**Mean (SD)**	**N**	**Mean (SD)**	**N**		
**Maternal biochemistry at 36 weeks' gestation**						
Fasting glucose (mmol/L)	4·42 (0·48)	151	4·35 (0·45)	143	−0·060 (−0·163 to 0·043)	0·25
2 h glucose (mmol/L)*	5·96 (1·46)	148	5·70 (1·32)	142	−0·251 (−0·565 to 0·062)	0·12
Fasting insulin (pmol/L)	208·98 (91·12)	131	227·73 (170·50)	127	1·005 (0·901 to 1·120)	0·93
HOMA-IR score†	5·98 (2·89)	131	6·30 (4·78)	123	0·974 (0·865 to 1·097)	0·67
C-reactive protein (mg/L)	9·20 (7·10)	150	7·47 (4·62)	140	0·860 (0·743 to 0·996)	0·04
Cholesterol (mmol/L)	6·32 (1·44)	144	6·33 (1·74)	139	1·004 (0·954 to 1·056)	0·88
HDL (mmol/L)	1·70 (0·38)	145	1·76 (0·43)	138	0·051 (−0·040 to 0·142)	0·27
LDL (mmol/L)	3·57 (1·13)	126	3·77 (1·25)	118	1·064 (0·982 to 1·152)	0·13
Triglycerides (mmol/L)	2·79 (0·84)	146	2·76 (0·88)	140	0·993 (0·926 to 1·064)	0·83
Interleukin-6 (mmol/L)	3·86 (4·10)	131	2·93 (1·37)	127	0·847 (0·754 to 0·952)	0·01
Leptin (ng/mL)	105·0 (52·4)	131	106·6 (58·8)	127	1·005 (0·902 to 1·120)	0·93
Serum cortisol (nmol/L)	821·7 (232·9)	131	867·0 (225·5)	127	1·062 (0·999 to 1·128)	0·05
NEFA (mmol/L)	0·47 (0·18)	131	0·46 (0·19)	127	0·947 (0·859 to 1·044)	0·27
PAI-1 to PAI-2 ratio	3·20 (2·61)	131	2·97 (2·79)	128	0·913 (0·771 to 1·081)	0·29
**Cord-blood biochemical outcomes**						
Glucose (mmol/L)	3·89 (1·24)	79	4·06 (1·08)	74	1·067 (0·974 to 1·170)	0·16
Insulin (pmol/L)	76·05 (52·02)	47	79·24 (61·12)	57	1·060 (0·767 to 1·463)	0·72
HOMA-IR score†	1·92 (1·39)	38	1·91 (2·00)	41	1·012 (0·701 to 1·462)	0·95
C-reactive protein (mg/L)‡	4·32 (19·55)	78	2·36 (2·29)	73	..	0·74
**Anthropometric variables**						
Maternal weight gain during pregnancy (kg)	7·23 (4·91)	156	6·70 (6·00)	143	−0·680 (−1·863 to 0·503)	0·26
Ponderal index (mass [g]/height^3^ [cm])§	2·60 (0·41)	143	2·67 (0·50)	130	1·032 (0·996 to 1·069)	0·08

All variables, except for maternal glucose and HDL, and neonatal C-reactive protein, were log-transformed for the statistical analysis and converted back to original scale for this table. HOMA-IR=homeostatic model assessment of insulin resistance. NEFA=non-esterified fatty acids. PAI=plasminogen activator inhibitor.

* After a 75 g oral glucose challenge.

† Fasting glucose (mmol/L) x insulin (μIU/L)/22·5.

‡ Kruskal–Wallis non-parametric test used.

§ Livebirths only; we removed outliers outside SD 6 and log-transformed data for the statistical analysis; results were back-transformed for this table.

**Table 4:** Adverse outcomes

		**Placebo group**	**Metformin group**	**OR (95% CI)**	**p value**
Women or their babies with a recorded serious adverse event		41/222 (18%)	37/225 (16%)	0·869 (0·533–1·417)	0·57
Maternal delivery and postnatal					
	Any caesarean section in index pregnancy	76/222 (34%)	65/219 (30%)	0·811 (0·543–1·211)*	0·31
	Primary caesarean section	46/222 (21%)	42/219 (19%)	0·908 (0·569–1·449)	0·69
	Postpartum haemorrhage >1000 mL	21/216 (10%)	20/212 (9%)	0·967 (0·508–1·842)	0·92
	Preterm birth†	14/220 (6%)	18/214 (8%)	1·345 (0·651–2·777)	0·47
	Development of gestational diabetes‡	36/153 (24%)	26/142 (18%)	0·728 (0·414–1·283)	0·27
	Pregnancy induced hypertension	14/222 (6%)	21/221 (10%)	1·56 (0·772–3·152)	0·22
	Pre-eclampsia	3/222 (1%)	7/221 (3%)	2·39 (0·61–9·36)	0·21
Fetal and neonatal outcomes (livebirths only)					
	Admission to the neonatal unit	29/219 (13%)	14/213 (7%)	0·461 (0·236–0·899)*	0·02
	Congenital anomaly	8/217 (4%)	7/209 (3%)	0·905 (0·322–2·543)	0·85
	Neonatal death in the delivery room	0/220	0/214	..	..
	Neonatal death at a later stage	2/220 (1%)	1/214 (<1%)	..	1·00*§
	Incidence of low birthweight <10th percentile	11/220 (5%)	14/214 (7%)	1·330 (0·590–2·999)	0·49
	Incidence of low birthweight <3rd percentile	3/220 (1%)	3/214 (1%)	..	1·00§
Maternal symptoms up to 36 weeks' gestation¶					
	Taste disturbance	32/198 (16%)	25/199 (13%)	0·745 (0·424–1·311)	0·31
	Skin reactions	39/198 (20%)	36/199 (18%)	0·900 (0·545–1·489)	0·68
	Abdominal pain	42/198 (21%)	49/199 (25%)	1·213 (0·759–1·940)	0·42
	Flatulence	44/198 (22%)	51/199 (26%)	1·206 (0·760–1·915)	0·43
	Constipation	57/198 (29%)	57/199 (29%)	0·993 (0·643–1·534)	0·98
	Diarrhoea	37/198 (19%)	83/199 (42%)	3·113 (1·975–4·908)	<0·0001
	Nausea	79/198 (40%)	97/199 (49%)	1·432 (0·962–2·132)	0·08
	Vomiting	43/198 (22%)	63/199 (32%)	1·670 (1·064–2·621)	0·03
	Headache	66/198 (33%)	65/199 (33%)	0·970 (0·638–1·474)	0·89

Data are n/N (%), unless otherwise indicated. OR=odds ratio.

* Post-hoc analysis.

† Livebirths only; four (29%) of 14 preterm births in the placebo group and three (17%) of 18 in the metformin group were spontaneous preterm births after preterm labour.

‡ International Association of Diabetes and Pregnancy Study Groups criteria: fasting glucose of 5·1 mmol/L or more or 2 h glucose of 8·5 mmol/L or more at either 28 or 36 weeks.

§ Fisher's exact test reported.

¶ For all symptoms, categories are none, mild, moderate, or severe. If a participant had any mild, moderate, or severe symptom at any time, this was recorded as “yes” for presence of maternal symptoms.
