# Supplementary material for: Effect of metformin on maternal and fetal outcomes in obese pregnant women (EMPOWaR): a randomised, double-blind, placebo-controlled trial
Source: Lancet Diabetes Endocrinol. 2015 Oct;3(10):778–86. doi: 10.1016/S2213-8587(15)00219-3 (PMC4673088; doi:10.1016/S2213-8587(15)00219-3)
Supplement: Supplementary appendix [file mmc1.pdf]

THE LANCET Diabetes & Endocrinology

Supplementary appendix

This appendix formed part of the original submission and has been peer reviewed. We post it as supplied by the authors.

Supplement to: Chiswick C, Reynolds RM, Denison F, et al. Effect of metformin on maternal and fetal outcomes in obese pregnant women (EMPOWaR): a randomised, double-blind, placebo-controlled trial. *Lancet Diabetes Endocrinol* 2015; published online July 10. http://dx.doi.org/10.1016/S2213-8587(15)00219-3.

Supplementary 1: Glucose, insulin and insulin resistance at 28 weeks

	Placebo		Metformin				
	**Mean (n)**	**SD**	**Mean (n)**	**SD**	**Adjusted mean difference/ratio**	**95 % CI**	**p-value**
Fasting glucose (mmol/L)	4.49 (184)	0.47	4.38 (175)	0.41	-0.105	-0.193, -0.016	0.021
2h glucose (mmol/L) [1]	5.85 (184)	1.20	5.58 (174)	1.32	-0.250	-0.504, 0.005	0.055
Fasting insulin (pmol/L) [2]	190.92 (154)	99.17	182.72 (144)	132.30	0.913	0.828,1.007	0.067
HOMA-IR score[3]	5.56 (153)	3.30	5.23 (144)	4.17	0.895	0.803,0.998	0.047

[1] After a 75g oral glucose challenge
[2] This parameter was log-transformed for the statistical analysis, and results back transformed for this table
[3] Fasting glucose (in mmol/l) x insulin (µIU/ml)/22.5. This parameter was log-transformed for the statistical analysis, and results back transformed for this table

Supplementary 2. Maternal anthropometry at 36 weeks and 3 months post-partum

	36 Weeks				3 months Post-Partum			
	Placebo		Metformin		Placebo		Metformin	
	Mean (n)	SD	Mean (n)	SD	Mean (n)	SD	Mean (n)	SD
Height (cm)	166.0 (153)	6.0	166.3 (142)	5.6	165.3 (125)	5.9	166.1 (127)	5.8
BMI calculated	40.4 (153)	5.4	40.6 (141)	4.9	37.4 (124)	5.2	38.3 (124)	5.6
Waist (cm)	120.0 (155)	13.2	119.0 (142)	11.1	109.2 (124)	12.8	109.9 (125)	13.9
Hip (cm)	130.1 (155)	12.3	131.3 (142)	11.8	127.3 (124)	12.2	128.6 (125)	13.4
Mid arm (cm)	36.5 (154)	4.9	36.5 (142)	4.4	37.1 (123)	4.7	37.4 (125)	4.4
Mid thigh (cm)	65.3 (154)	7.4	65.2 (139)	6.8	64.3 (122)	6.7	65.8 (124)	6.8
Tricep skinfold (mm)	30.4 (155)	10.3	31.3 (143)	12.0	32.2 (123)	10.8	33.4 (125)	11.4
Bicep skinfold (mm)	26.0 (155)	10.5	26.9 (143)	11.6	27.2 (123)	12.1	29.7 (125)	15.1
Subscapular skinfold (mm)	32.7 (154)	13.5	34.5 (141)	13.9	33.2 (123)	13.1	35.9 (124)	13.2
Maternal % fat [4]	46.3 (31)	4.84	47.8 (30)	4.63	47.45 (29)	4.97	48.35 (30)	5.31
Weight gain during pregnancy (kg) [5]	7.23 (156)	4.91	6.70 (143)	6.00	-0.13 (124)	6.22	0.07 (124)	9.82
**Neonatal outcomes (live births only)**	**At or shortly after birth**				**At 3 months post partum**			
	**Placebo**		**Metformin**		**Placebo**		**Metformin**	
	**Mean (n)**	**SD**	**Mean (n)**	**SD**	**Mean (n)**	**SD**	**Mean (n)**	**SD**
Age at which measurements made (days)	1.04 (157)	2.44	0.97 (145)	2.44	99.59 (128)	13.12	97.72 (129)	14.01
Length (cm)[7]	51.2 (150)	4.0	50..73 (139)	3.3	62.13 (124)	4.38	61.69 (125)	6.33

[4] Measured only in Edinburgh participants
[5] Summary stats at week 36 are a repeat from Table 3, presented here for completeness.

Head circumference (cm)	34.7 (164)	4.2	34.8 (152)	3.6	41.30 (124)	2.87	41.02 (122)	4.42
Ponderal index (mass [g] / height ^{3} [cm]) [6] [7]	2.60 (143)	0.41	2.67 (130)	0.50	2.58 (124)	0.82	2.52 (124)	1.00
Tricep skinfold thickness (mm)	14.3 (111)	20.6	16.4 (99)	27.9	22.05 (106)	10.40	24.61 (104)	11.00
Subscapular skinfold (mm)	13.5 (113)	20.4	15.7 (98)	28.0	17.00 (104)	23.95	23.11 (104)	31.33
Baby % fat [8]	12.1 (22)	5.7	12.9 (21)	4.5	25.88 (31)	6.13	23.19 (29)	5.91
Weight at this time (g)[7] [9]	3502.65 (163)	561.32	3455.18 (146)	545.08	6085.04 (128)	1276.59	5971.97 (132)	1724.20

[6] Summary stats at birth are a repeat from Table 3, presented here for completeness.
[7] Outliers outside +/- 6SD were removed.
[8] Measured only in Edinburgh participants
[9] Baby weight was recorded on two occasions – at birth by delivery team (figure used for z-score calculations) and then at time of taking research measurements by research team (this second figure is shown here and is used for calculation of ponderal index).

Supplementary 3. Metabolic and inflammatory markers at 28 weeks

	Placebo		Metformin	
	**Mean (n)**	**SD**	**Mean (n)**	**SD**
CRP (mg/L)	10.65 (176)	7.41	9.78 (164)	6.54
IL-6 (mmol/L)	2.73 (154)	2.16	2.38 (144)	1.19
Leptin (ng/ml)	104.4 (154)	46.4	102.3 (144)	50.5
Serum cortisol (nmol/L)	716.5 (154)	230.8	777.2 (144)	252.8
NEFA (mmol/L)	0.42 (154)	0.14	0.43 (144)	0.16

Supplementary Table 4 Baseline Characteristics (per protocol analysis)

	Placebo		Metformin	
	n=118		n=109	
**Demographics and lifestyle (participant)**	**Mean or number (n)**	**SD or %**	**Mean or number (n)**	**SD or %**
Age (years)	29.6	5.0	29.8	5.6
Current smoking	13	11.0%	13	11.9%
Current alcohol	6	5.1%	0	0%
Illicit drug users	0	0%	0	0%
Highest educational qualification				
school up to 16 years or less	37	31.4%	26	23.9%
school 16 years or higher	81	68.6%	83	76.1%
At least one previous pregnancy ≥12 weeks gestation	87	73.7%	68	62.4%
Systolic BP (mmHg)	119.3	11.2	117.1	11.3
Diastolic BP (mmHg)	69.0	7.7	68.5	7.9
Gestation at baseline (days)	98.9.	9.0	100.0	7.9

**Medical history (participant)**				
Pre-eclampsia or pregnancy induced hypertension	3	2.5%	6	5.5%
Pre- pregnancy hypertension requiring treatment	1	0.8%	1	0.9%
PCOS	14	11.9%	16	14.7%
Depression requiring treatment	33	28.0%	24	22.0%
Anxiety requiring treatment	7	5.9%	7	6.4%

**Family history (participant)**				
Cardiovascular disease	41	34.7%	31	28.4%
Pre-eclampsia	8	6.8%	4	3.7%
Diabetes	54	45.8%	47	43.1%
Other	58	49.2%	57	52.3%
**Anthropometry (participant)**				
Height (cm)	166.1	6.0	165.8	5.7
Weight (kg)	103.7	17.0	104.0	15.2
BMI calculated (kg/m^2)	37.5	5.5	37.8	4.7
Waist (cm)	108.3	12.6	108.6	11.2
Hip (cm)	126.8	11.6	127.5	12.2
Mid arm (cm)	36.6	4.7	37.1	4.4
Mid-thigh (cm)	64.2	7.3	65.3	7.0
Tricep skinfold (mm)	33.3	9.4	32.6	9.7
Bicep skinfold (mm)	27.4	10.1	27.8	10.7
Subscap skinfold (mm)	35.3	11.0	34.8	11.7
Maternal % fat[10]	46.2	5.2	48.6	5.0

[10] Measured only in Edinburgh participants

Blood tests recruitment visit (participant)				
Fasting glucose (mmol/L)	4.42	0.36	4.41	0.37
2h glucose (mmol/L)[11]	5.54	1.18	5.17	1.10
Fasting insulin (pmol/L)	159.46 (101)	72.64	152.23 (92)	62.44
HOMA-IR score[12]	4.59 (101)	2.32	4.34 (92)	1.82
CRP (mg/L)	11.4	7.9	10.0	6.3
Cholesterol (mmol/L)	4.86 (117)	1.16	4.82 (108)	1.13
HDL (mmol/L)	1.67 (117)	0.38	1.64 (108)	0.39
LDL (mmol/L)	2.98 (106)	0.75	2.90 (101)	0.90
Triglycerides (mmol/L)	1.51 (117)	0.54	1.45 (108)	0.58
IL-6 (mmol/L)	2.30 (101)	1.12	2.03 (92)	1.11
Leptin (ng/ml)	90.7 (118)	46.2	99.8 (109)	39.2
Serum cortisol (nmol/L)	384.8 (101)	135.5	438.2 (92)	186.5
NEFA (mmol/L)	0.54 (101)	0.20	0.47 (92)	0.16
PAI1/PAI2 ratio	1.55 (91)	1.6	2.16 (82)	6.49

Putative father details				
Height (cm)	178.5	7.8	177.9	13.2
Weight (kg)	92.1	21.9	94.6	27.7
Ethnicity				

[11] After a 75g oral glucose challenge
[12] Fasting glucose (in mmol/l) x insulin (in µIU/ml)/22.5

Caucasian	114	96.6%	101	92.7%
Mixed	1	0.8%	2	1.8%
Asian	0	0%	2	1.8%
Black	2	1.7%	3	2.8%
Chinese	0	0%	0	0%
Other	1	0.8%	1	0.9%

Supplementary Table 5. Primary outcome (per protocol analysis)

	Placebo		Metformin				
**Primary outcome**	**Mean (n)**	**SD**	**Mean (n)**	**SD**	**Adjusted mean difference**	**95 % CI**	**p-value**
Z score of birthweight centile[13]	0.3130 (117)	0.9781	0.3604 (108)	1.0580	0.068	-0.188, 0.324	0.60
**Birth outcome (all births)**	**Mean or number (n)**	**SD or %**	**Mean or number (n)**	**SD or %**	**OR[14]**	**95 % CI**	**p-value**
Live birth at ≥ 24 weeks gestation	117 (118)	99.2%	108 (108)	100%			
Stillbirth at ≥ 24 weeks gestation, miscarriage or termination of pregnancy	1 (118)	0.8%	0	0%	<0.001	<0.001, >999.999	0.96
**Birth outcome (liveborn babies at ≥ 24 weeks gestation)**							
Gestational age at delivery (days)	277.6 (117)	12.7	276.6 (108)	11.5			
Male sex	58 (118)	49.2%	54 (108)	50%			
Birthweight at delivery (g)	3539.0 (117)	553.9	3503.6 (108)	562.8			
Birthweight centile	58.527 (117)	27.7	59.894 (108)	28.3			

[13] Centile by gestational age, sex and parity for live births at ≥ 24 weeks gestation
[14] Post hoc analysis

Supplementary Table 6. Secondary outcomes (per protocol analysis)

	Placebo		Metformin				
	Mean (n)	SD	Mean (n)	SD	Adjusted mean difference/ratio	95 % CI	p-value
**Maternal biochemistry at 36 weeks gestation**							
Fasting glucose (mmol/l)	4.43(104)	0.51	4.34(93)	0.45	-0.091	-0.221, 0.040	0.17
2h glucose (mmol/l) [15]	6.04(103)	1.53	5.79(92)	1.34	-0.248	-0.643, 0.148	0.22
Fasting insulin (pmol/L)	221.48(88)	93.06	226.34(79)	181.06	0.939	0.819, 1.075	0.36
HOMA-IR score[16]	6.36(88)	2.96	6.22(77)	4.90	0.912	0.784, 1.060	0.23
CRP (mg/L)	8.91(104)	6.39	7.48(93)	4.58	0.901	0.760, 1.070	0.23
Cholesterol (mmol/L)	6.29(100)	1.54	6.16(91)	1.88	0.974	0.913, 1.039	0.42
HDL (mmol/L)	1.71(100)	0.37	1.76(91)	0.38	0.055	-0.046, 0.155	0.29
LDL (mmol/L)	3.67(89)	1.09	3.71(80)	1.22	1.013	0.923, 1.113	0.78
Triglycerides (mmol/L)	2.79(101)	0.90	2.84(92)	0.96	1.031	0.942, 1.127	0.51
IL-6 (mmol/L)	3.66(88)	3.73	2.77(79)	1.26	0.858	0.745, 0.988	0.03
Leptin (ng/ml)	103.80(88)	55.34	101.26(79)	47.02	1.007	0.886, 1.145	0.92
Serum cortisol (nmol/L)	806.48(88)	225.00	888.39(79)	250.73	1.092	1.010, 1.181	0.03
NEFA (mmol/L)	0.47(88)	0.19	0.48(79)	0.21	1.041	0.919, 1.179	0.52
PAI1/PAI2 ratio	3.40(91)	2.65	3.31(82)	3.09	0.895	0.721, 1.113	0.32
**Cord blood biochemical outcomes**							
Glucose (mmol/l)	3.94(62)	1.25	4.02(54)	1.05	1.062	0.955, 1.181	0.26

[15] After a 75g oral glucose challenge
[16] Fasting glucose (in mmol/l) x insulin (μIU/ml)/22.5

Insulin (pmol/L)	77.37(37)	51.95	83.62(45)	63.96	1.137	0.805, 1.607	0.46
HOMA-IR score[16]	1.83(32)	1.36	1.93(30)	2.19	1.066	0.720, 1.579	0.74
CRP (mg/L) [17]	4.85(62)	21.89	2.15(53)	1.82			0.80
Anthropometric variables							
Maternal weight gain during pregnancy (kg)	7.40(106)	4.56	6.85(93)	6.11	-0.339	-1.769, 1.091	0.64
Ponderal index (mass [g] / height^3 [cm]) (live births only) [18]	2.64(90)	0.42	2.63(79)	0.46	1.004	0.961, 1.049	0.85

[17] Kruskal–Wallis non parametric test used
[18] Outliers outside +/- 6SD were removed, and data log-transformed for the statistical analysis, and results back transformed for this table.
Note, all parameters with the exception of maternal glucose and HDL, and neonatal CRP were log-transformed for the statistical analysis, and converted back to original scale for this table

Supplementary Table 7. Adverse outcomes (secondary outcomes)(per protocol analysis)

	Placebo		Metformin				
	**Number (n)**	**%**	**Number (n)**	**%**	**OR**	**95 % CI**	**p-value**
Women or their babies with a recorded serious adverse event	22 (118)	18.6	14 (109)	12.8	0.643	0.311, 1.331	0.28
**Maternal delivery and postnatal**							
Any caesarean section in index pregnancy	43 (118)	36.4	31 (108)	28.7	0.702	0.401, 1.230	0.26
Primary caesarean section	25 (118)	21.2	22 (108)	20.4	0.952	0.500, 1.811	1.0000
Postpartum haemorrhage > 1000ml	13 (118)	11.4	9 (109)	8.5	0.721	0.295, 1.763	0.51
Preterm birth[19]	4 (117)	3.4	8 (108)	7.4	2.260	0.661, 7.732	0.24
Development of gestational diabetes[20]	22 (104)	21.2	15 (92)	16.3	0.726	0.351, 1.501	0.39
Pregnancy induced hypertension[21]	11 (118)	9.3%	11 (109)	10.1%	1.092	0.453, 2.631	0.84
Pre-eclampsia[21]	3 (118)	2.5%	3 (109)	2.8%	1.085	0.214, 5.493	0.92
**Fetal and neonatal outcomes (live births only)**							
Admission to the neonatal unit	13 (116)	11.2	8 (108)	7.4	0.634	0.252, 1.595	0.33
Congenital anomaly	4 (115)	3.5	4 (107)	3.7	1.078	0.263, 4.421	0.92
Neonatal death in the delivery room	0 (117)	0	0 (108)	0			
Neonatal death at a later stage	0 (117)	0	0 (108)	0			
Incidence of low birthweight <10th centile	6 (117)	5.1	6 (108)	5.6	1.088	0.340, 3.482	0.89
Incidence of low birthweight <3rd centile[22]	1 (117)	0.9	1 (108)	0.9			1.0000

[19] Live births only; 4/14 preterm births in the placebo group and 3/18 in the metformin group were spontaneous preterm births following preterm labour.
[20] IADPSG criteria: Fasting glucose >= 5.1 mmol/l or 2hr glucose >= 8.5 mmol/l on either 28 and 36 weeks
[21] As defined by the local investigator
[22] Fisher's exact test reported

	Placebo		Metformin				
	n=118		n=109				
**Maternal symptoms up to 36 week gestation** [23]							**p-value**
Taste disturbance	20	16.9	17	15.6	0.905	0.447, 1.835	0.78
Skin reactions	23	19.5	21	19.3	0.986	0.510, 1.905	0.97
Abdominal pain	26	22.0	32	29.4	1.471	0.807, 2.678	0.21
Flatulence	28	23.7	38	34.9	1.720	0.964, 3.069	0.07
Constipation	38	32.2	37	33.9	1.082	0.622, 1.882	0.78
Diarrhoea	24	20.3	60	55.0	4.896	2.669, 8.617	<0.0001
Nausea	46	39.0	49	45.0	1.278	0.754, 2.168	0.36
Vomiting	24	20.3	34	31.2	1.775	0.970, 3.249	0.06
Headache	40	33.4	37	33.9	1.002	0.578, 1.737	0.99

[23] For all symptoms, categories are none/mild/moderate or severe. If a participant had any symptom mild, moderate or severe, at any time this is recorded as "yes".
